# Detection of Mild Cognitive Impairment From Non-Semantic, Acoustic Voice Features: The Framingham Heart Study

**DOI:** 10.2196/55126

**Published:** 2024-08-22

**Authors:** Huitong Ding, Adrian Lister, Cody Karjadi, Rhoda Au, Honghuang Lin, Brian Bischoff, Phillip H Hwang

**Affiliations:** 1 Department of Anatomy and Neurobiology Boston University Chobanian & Avedisian School of Medicine Boston, MA United States; 2 The Framingham Heart Study Boston University Chobanian & Avedisian School of Medicine Boston, MA United States; 3 Headwaters Innovation, Inc. Inver Grove Heights, MN United States; 4 Department of Epidemiology Boston University School of Public Health Boston, MA United States; 5 Slone Epidemiology Center and Departments of Neurology and Medicine Boston University Chobanian & Avedisian School of Medicine Boston, MA United States; 6 Department of Medicine University of Massachusetts Chan Medical School Worcester, MA United States

**Keywords:** early detection, Alzheimer disease and related dementias, mild cognitive impairment, digital voice, machine learning, smartphone, mobile phone

## Abstract

**Background:**

With the aging global population and the rising burden of Alzheimer disease and related dementias (ADRDs), there is a growing focus on identifying mild cognitive impairment (MCI) to enable timely interventions that could potentially slow down the onset of clinical dementia. The production of speech by an individual is a cognitively complex task that engages various cognitive domains. The ease of audio data collection highlights the potential cost-effectiveness and noninvasive nature of using human speech as a tool for cognitive assessment.

**Objective:**

This study aimed to construct a machine learning pipeline that incorporates speaker diarization, feature extraction, feature selection, and classification to identify a set of acoustic features derived from voice recordings that exhibit strong MCI detection capability.

**Methods:**

The study included 100 MCI cases and 100 cognitively normal controls matched for age, sex, and education from the Framingham Heart Study. Participants' spoken responses on neuropsychological tests were recorded, and the recorded audio was processed to identify segments of each participant's voice from recordings that included voices of both testers and participants. A comprehensive set of 6385 acoustic features was then extracted from these voice segments using OpenSMILE and Praat software. Subsequently, a random forest model was constructed to classify cognitive status using the features that exhibited significant differences between the MCI and cognitively normal groups. The MCI detection performance of various audio lengths was further examined.

**Results:**

An optimal subset of 29 features was identified that resulted in an area under the receiver operating characteristic curve of 0.87, with a 95% CI of 0.81-0.94. The most important acoustic feature for MCI classification was the number of filled pauses (importance score=0.09, *P*=3.10E–08). There was no substantial difference in the performance of the model trained on the acoustic features derived from different lengths of voice recordings.

**Conclusions:**

This study showcases the potential of monitoring changes to nonsemantic and acoustic features of speech as a way of early ADRD detection and motivates future opportunities for using human speech as a measure of brain health.

## Introduction

Alzheimer disease and related dementias (ADRDs) constitute a significant public health issue, impacting an estimated 6.2 million individuals in the United States, with projections indicating the number of cases to grow to 12.7 million and 150 million globally by 2050 [1,2]. Emerging evidence suggests that the functional, psychological, pathological, and physiological alterations associated with ADRD may manifest many years prior to the clinical onset of cognitive dysfunction [3-6]. This increasing awareness has sparked interest in early detection and monitoring of ADRD, with the goal of implementing timely preventive and therapeutic strategies to slow the progression of the disease. As effective as they are in identifying individuals at high risk of ADRD, conventional diagnostic methods, such as cerebrospinal fluid biomarkers and neuroimaging, face accessibility limitations primarily due to their high costs [7] and high subject burden. This limits their applicability to other groups, particularly populations in lower-resourced settings, in effectively monitoring the dynamic progression of the disease. Therefore, there is an urgent need for an effective detection method that has a much broader and more inclusive reach for the early detection of ADRD.

Producing speech is a cognitively complex task that engages various cognitive domains [8], and the ease of audio data collection underscores the potential cost-effectiveness and noninvasiveness that using human speech-based features may offer to facilitate early identification of cognitive impairment, including mild cognitive impairment (MCI). Studies have indicated that language deficits may manifest in the prodromal stages of cognitive impairment, often years before the clinical diagnosis of dementia [9,10]. Speech, however, is far richer in characterizing cognition than just language. Audio recordings can yield a variety of attributes, encompassing both acoustic and linguistic features. Acoustic features, given their language independence, have the potential for broader global applicability. Previous studies from the Framingham Heart Study (FHS) demonstrated significant associations between acoustic features extracted from voice recordings and 2 primary clinical indices of neurodegeneration: neuropsychological (NP) test performance [11] and brain volumes [12]. Moreover, acoustic-based models can be readily deployed on devices such as hand-held recorders, smartphones, tablets, and other internet-connected mobile devices, enabling widespread usage. These characteristics enable voice as a potential digital biomarker for early cognitive impairment monitoring and detection of MCI.

While the use of speech recordings as a novel measure of cognition is still in the early stages of validation, most of the previous studies have relied on a limited set of acoustic features [13-16], potentially constraining the enhancement of early detection capabilities for ADRD. For instance, some studies have concentrated on Mel-frequency cepstral coefficients [13,15], while others have explored a narrow range of temporal and spectral features (such as duration of utterance, number and length of pauses, and F0) [14,16]. There has been a notable absence of exploration into diverse categories of features, including energy, spectral, cepstral, and voicing-related features. Although deep learning has been used to investigate these features, its complexity often compromises interpretability. Therefore, there is a need for research to use more interpretable methods for exploring a richer set of acoustic features for the detection of MCI. Furthermore, the question of whether extensive voice recordings are necessary to achieve better cognitive assessment performance has not been thoroughly investigated. These issues have significant implications for the widespread, real-world application of speech as a digital data modality for cognitive assessment.

Therefore, the aims of this study were to explore the utility of acoustic features derived from human speech for the identification of MCI and to assess the impact of the duration of voice recordings on the predictive performance of MCI identification.

## Methods

### Study Population

Initiated in 1948, FHS is a community-based, longitudinal cohort study. This study initially included 605 FHS participants with at least one audio recording who were aged 60 years or older at the time of the NP exam visit where the recordings were collected. Then, a case-control data set was created consisting of 100 MCI cases and 100 cognitively normal (CN) controls and matched on age, sex, and education to control for potential confounders and ensure the reliability of the study results. MCI cases were identified through a clinical review conducted by a panel including at least one neurologist and one neuropsychologist based on criteria from the *DSM-IV* (*Diagnostic and Statistical Manual of Mental Disorders* [Fourth Edition]) and the National Institute of Neurological Disorders and Stroke–Alzheimer Disease and Related Disorders [17]. The details of the cognitive status determination can be found in previous studies [15]. The participants were stratified into 6 age groups, with each group spanning a 5-year interval from 60 to 89 years (eg, 60-64, 65-69, 70-74, 75-79, 80-84, and 85-89 years). Additionally, there was a separate category for individuals aged 90 years and older. Study participants were also stratified into 4 education groups: high school nongraduates, high school graduates, individuals with some college education, and college graduates. Subsequently, controls were selected from the data set who matched the cases based on age, sex, and education. The earliest collected voice recording from each participant was included in this analysis.

### Ethical Considerations

The procedures and protocols of the FHS were approved by the institutional review board of the Boston University Medical Campus (FHS is H-32132), and written informed consent was obtained from all participants.

### Voice Recordings

FHS has been monitoring cognitive status since 1976, which includes comprehensive NP testing [18]. Since 2005, FHS has digitally recorded all responses to NP test questions that required a voice response, which encompasses the spoken interactions between the tester and the participant. These recordings have been stored in the .wav format and downsampled to 16 kHz. This study included digital voice recordings between September 2005 and March 2020.

### Machine Learning Pipeline

**Figure 1:**
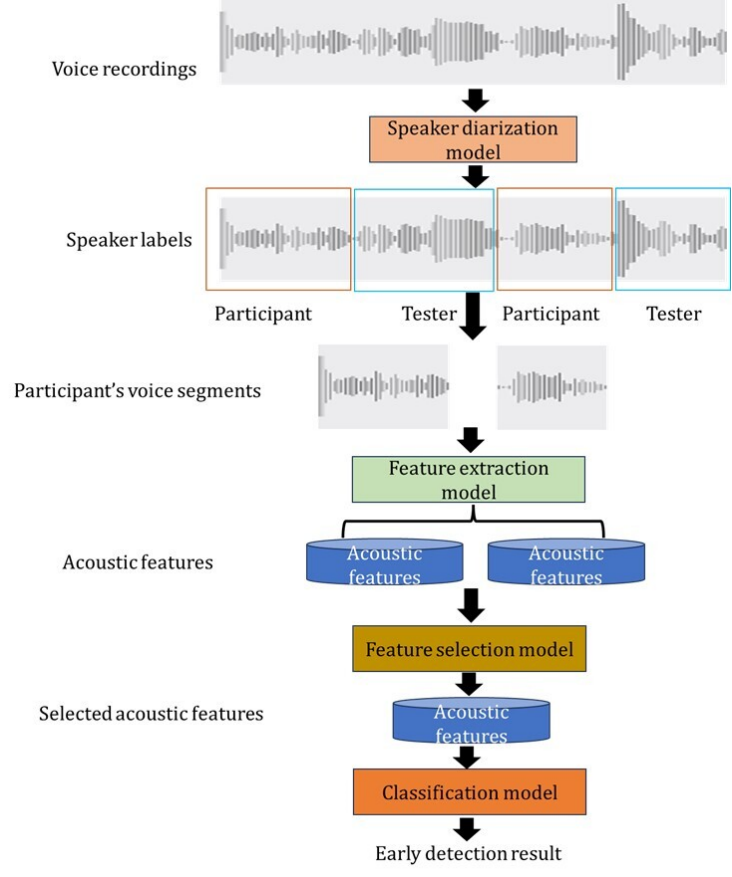
The machine learning pipeline for MCI detection from voice recordings.

This study developed a machine learning pipeline that incorporated speaker diarization, feature extraction, feature selection, and classification to identify a set of acoustic features that exhibited strong MCI detection capability (Figure 1).

#### Speaker Diarization

To accurately analyze the speech of the participants, it is crucial to distinguish between the participant and the tester and to determine “who spoke when” [19]. This process is known as speaker diarization, which involves segmenting the voice recordings based on the speaker's identity. In this study, the open-source speaker diarization package, pyannote, was used to automatically segment each recording into hypothesized utterances from the tester and the participant [20,21]. Since the NP administration testing process in FHS is standardized, the segmented dominant speaker, based on the duration of the voice recording, was labeled as the participant's speech in this study. These participant segments were combined for subsequent analysis.

#### Feature Extraction

To extract relevant information from the voice recordings, OpenSMILE software (version 2.1.3; audEERING) [22] and Praat software (University of Amsterdam) [23] were used, which facilitated the extraction of a comprehensive set of 6376 features [24] and 9 features, respectively. The OpenSMILE feature set used in this study consisted of 65 low-level descriptors (LLDs). These descriptors included energy, spectral, cepstral, and voicing-related features. Each recording was divided into segments of 20 milliseconds using a sliding window approach with a shifting size of 10 milliseconds [25,26]. The LLDs were extracted from each segment. By allowing for overlaps between successive windows, we were able to facilitate the conservation of information continuity and enable a more precise capture of the signal's dynamics [25,26]. First-order delta regression coefficients were calculated for all LLDs. A comprehensive set of functionals, such as mean, maximum, minimum, SD of segment length, and linear regression slope, were applied to extract statistical characteristics from the LLDs and deltas over the full recordings [27-29]. This process provided a concise representation of the acoustic features across the entire recording. As a result of this summarization process, each recording was represented by a set of 6376 features from OpenSMILE, capturing essential information about the acoustic properties of the audio data. The details of the feature generation process can be found in a prior study [30]. The Praat script was used to generate 9 features on syllable nuclei and fill pauses in the voice recordings [31].

#### Feature Selection

First, *z* scores were computed for each feature, and those with an absolute *z* score greater than 2 were removed as they were considered as outliers. Then, *t* tests (2-tailed) were used to determine whether there was a significant difference in each feature between the MCI and CN groups. Features that exhibited a significant difference below a *P* value threshold of .002 were then selected to be included in the model.

#### Classification Model

A random forest model was built using a final set of 29 selected features, and the performance of the model was evaluated using 10-fold cross-validation. To evaluate the MCI detection performance of the model, the area under the receiver operating characteristic curve (AUC), along with the 95% CI, for the random forest algorithm was obtained. The importance of each feature was computed using an impurity-based approach [32].

#### Comparison of Performance Across Different Audio Recording Lengths

To investigate the impact of the length of the audio recordings on the MCI classification performance, the first 5, 10, 15, and 30 minutes of the whole recording for each participant were extracted. Subsequently, the same processing steps were applied to each extracted audio segment, including speaker diarization, feature extraction, and the construction of the MCI classification model.

## Results

### Cohort Descriptive

The study sample included 200 participants, of whom 100 were diagnosed with MCI and the other 100 were classified as CN. In the overall sample, the average age was 74 (SD 6) years, and 46% (92/200) were female, with the sex distribution (females versus males) equal in both MCI and CN groups. Education in the overall sample was distributed as follows: 18 participants (18/200, 9%) did not graduate from high school, 54 participants (54/200, 27%) were high school graduates, 66 participants (66/200, 33%) had completed some college, and 62 participants (62/200, 31%) held at least a college degree.

### Feature Selection and Detection Performance

**Figure 2:**
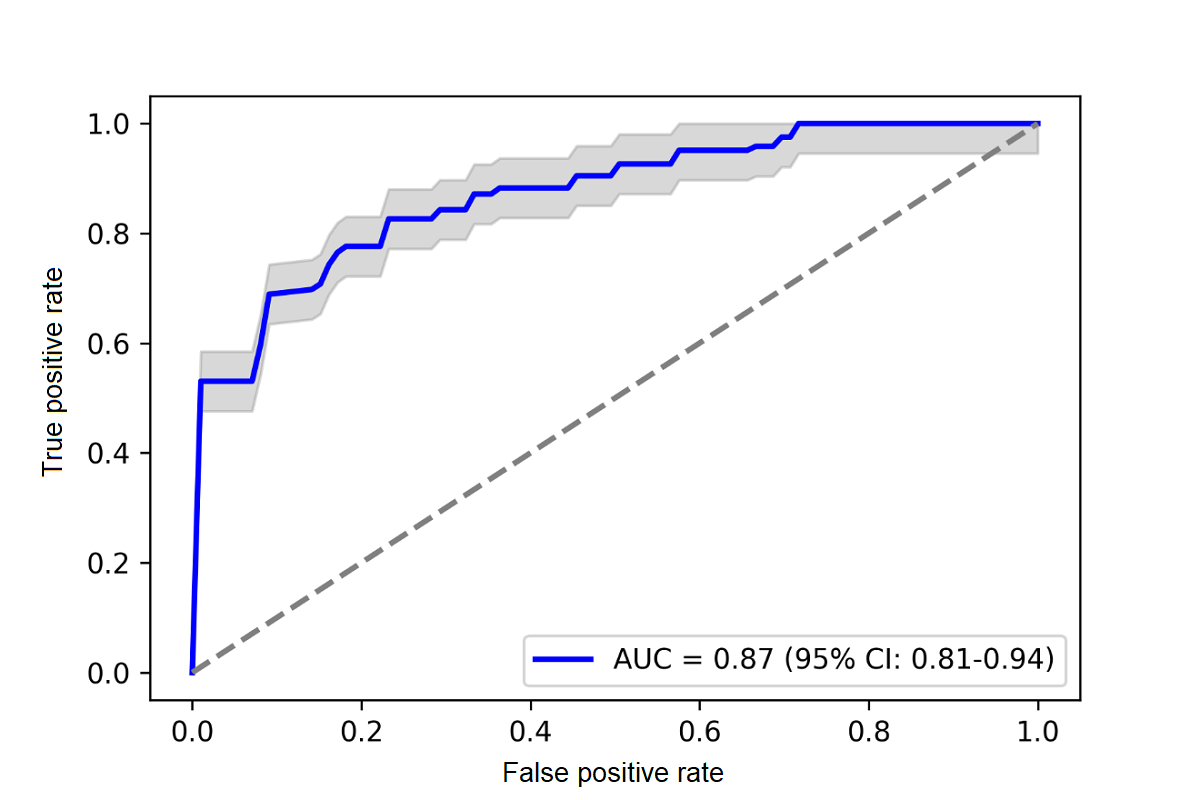
Receiver operating characteristic (ROC) curve of the random forest model for MCI classification. The mean ROC is depicted by the blue line, while the shaded gray area surrounding the curve represents confidence intervals, offering insights into the associated uncertainty of the curve.

Table 1 presents the 29 acoustic features significantly associated with cognitive status, selected using a *P* value threshold of .002. The table also displays the importance scores of these features for the classification of MCI, with higher values indicating greater importance. The most important acoustic feature for MCI classification was the number of filled pauses, with an importance score of 0.09. The optimal model was achieved when including these 29 acoustic features that were based on using a *z* score cutoff of 2 and a *P* value threshold of .002 (AUC 0.87, 95% CI 0.81-0.94; Figure 2).

**Table 1:** The optimal acoustic feature set for mild cognitive impairment detection.

Feature	Description	Importance^a^	*P* value^b^
nrFP	Number of filled pauses	0.09	<.001
tFP	Total time of filled pauses	0.08	<.001
mfcc_sma[11]_meanFallingSlope	Mean of the falling slope of the second MFCC^c^	0.06	.001
pcm_fftMag_spectralHarmonicity_sma_risetime	Rise time of the signal for magnitude of psychoacoustic harmonicity	0.05	.001
mfcc_sma[14]_risetime	Rising time of the second MFCC	0.05	.001
pcm_fftMag_spectralRollOff90.0_sma_de_minPos	Absolute position of the minimum value of the deltas of magnitude of the spectral roll-off point 90%	0.05	<.001
mfcc_sma_de[9]_upleveltime25	Percentage of time over 25% of the range of variation of the deltas of the ninth MFCC	0.05	<.001
audSpec_Rfilt_sma[25]_quartile1	First quartile of the RASTA-style filtered auditory spectrum, band 25	0.04	.002
mfcc_sma[1]_segLenStddev	Standard deviation of the segment lengths of the first MFCC	0.04	.002
audSpec_Rfilt_sma_de[5]_iqr2-3	Interquartile 2-3 of the deltas of the RASTA-style filtered auditory spectrum, band 5	0.04	<.001
pcm_fftMag_fband250-650_sma_de_stddev	Standard deviation of the delta of magnitude of the frequency band 250-650 Hz	0.04	.002
mfcc_sma_de[2]_lpc1	Linear prediction coefficient as one of the deltas of the second MFCC	0.04	.002
pcm_fftMag_fband250-650_sma_de_rqmean	Root-quadratic mean of the deltas of magnitude of the frequency band 250-650 Hz	0.04	.002
audSpec_Rfilt_sma[7]_upleveltime75	Percentage of time over 75% of the range of variation of the RASTA-style filtered auditory spectrum, band 7	0.03	.001
mfcc_sma[2]_maxSegLen	Maximum of the segment lengths of the second MFCC	0.03	.002
audSpec_Rfilt_sma_de[5]_upleveltime75	Percentage of time over 75% of the range of variation of the deltas of the RASTA-style filtered auditory spectrum, band 5	0.03	.002
audSpec_Rfilt_sma_de[5]_upleveltime90	Percentage of time over 90% of the range of variation of the deltas of the RASTA-style filtered auditory spectrum, band 5	0.03	.002
audSpec_Rfilt_sma_de[7]_upleveltime75	Percentage of time over 75% of the range of variation of the deltas of the RASTA-style filtered auditory spectrum, band 7	0.03	.002
audSpec_Rfilt_sma_de[15]_lpc0	Linear prediction coefficient zero of the delta of the RASTA-style filtered auditory spectrum, band 15	0.03	.002
audSpec_Rfilt_sma_de[15]_lpc1	Linear prediction coefficient one of the deltas of the RASTA-style filtered auditory spectrum, band 15	0.03	<.001
audSpec_Rfilt_sma_de[15]_lpc2	Linear prediction coefficient 2 of the delta of the RASTA-style filtered auditory spectrum, band 15	0.03	<.001
audSpec_Rfilt_sma[18]_qregc1	Quadratic regression coefficient 1 of the RASTA-style filtered auditory spectrum, band 19	0.03	<.001
audSpec_Rfilt_sma[18]_qregc2	Quadratic regression coefficient 2 of the RASTA-style filtered auditory spectrum, band 19	0.03	<.001
audSpec_Rfilt_sma_de[15]_lpc3	Linear prediction coefficient 3 of the delta of the RASTA-style filtered auditory spectrum, band 15	0.02	<.001
audspec_lengthL1norm_sma_peakRangeAbs	Absolute peak range of the sum of the auditory spectrum	0.02	.002
pcm_fftMag_spectralRollOff25.0_sma_pctlrange0-1	Outlier robust signal range “max-min” represented by the range of the 1% and the 99% percentile from the magnitude of the spectral roll-off point 25%	0.01	<.001
mfcc_sma_de[4]_peakMeanRel	Relative peak mean of the delta of the fourth MFCC	0.01	<.001
pcm_fftMag_spectralRollOff75.0_sma_quartile1	First quartile of magnitude of the spectral roll-off point 75%	0.00	<.001
pcm_fftMag_spectralRollOff75.0_sma_quartile3	Third quartile of magnitude of the spectral roll-off point 75%	0.00	<.001

^a^Importance was the impurity-based importance score of each acoustic feature that was computed as the mean of accumulation of the impurity decrease within each tree of the random forest.

^b^The *P* value was calculated using a *t* test (2-tailed) for each acoustic feature. Only the acoustic features with a *P* value less than .002 were included in the model.

^c^MFCC: Mel-frequency cepstral coefficient.

### Comparison of Performance Across Different Audio Recording Lengths

In addition to the optimal model based on whole recordings (1+ hour), we further examined the MCI detection performance of various audio recording lengths. In the case of 5-minute audio segments, we identified 21 acoustic features that exhibited significant associations with cognitive status (eg, *P*<.002). The random forest model constructed using these 21 features achieved an AUC of 0.79 (95% CI 0.73-0.86). Similarly, for the 10-minute audio segments, we identified 25 significant acoustic features and achieved an AUC of 0.81 (95% CI 0.75-0.87). When using 15-minute audio segments, 17 acoustic features were found to be significantly associated with cognitive status, leading to an AUC of 0.80 (95% CI 0.75-0.86) from the random forest model. Lastly, in the case of 30-minute audio segments, 17 acoustic features were significantly associated with cognitive status, and the random forest model achieved an AUC of 0.82 (95% CI 0.76-0.89). The accuracy, sensitivity, and specificity of these models were presented in Multimedia Appendix 1. These metrics were computed based on the means and SDs obtained using 10-fold cross-validation.

## Discussion

### Principal Findings

This study developed a machine learning pipeline to optimize the detection capability of acoustic features for MCI. We identified 29 acoustic features from 200 FHS participants’ voice recordings collected at their NP exams, which yielded an AUC of 87% in classifying those with normal cognition versus MCI. Our findings highlight the significant potential of acoustic-based features of human speech as an easily collectible and accurate data modality for early ADRD detection.

Detecting ADRD early in the disease course and implementing timely interventions to slow its progression continue to be the primary strategies for addressing this condition. The method developed in this study using acoustic features for MCI monitoring aligns well with this goal. Specifically, despite recent FDA approvals for aducanumab and lecanemab as disease-modifying treatments for ADRD, concerns have emerged about the inclusivity of the trial population and the equitable distribution of benefits to all potential beneficiaries [33]. The acoustic feature-based machine learning approach in this study addresses the limited early detection capability of traditional NP tests for asymptomatic individuals, as well as the challenges associated with the cost and time-consuming nature of cerebrospinal fluid and blood-based biomarkers [34]. Speech data collection presents a noninvasive and accessible approach for cognitive health monitoring. This motivates potential future applications where passive voice collection tools, like hearing aids, could be used to gather such data. The use of nonsemantic, acoustic features of speech offers practical advantages from the perspective of data privacy and security. Unlike linguistic features, which may raise concerns around individual privacy and confidentiality, acoustic features can be derived without the need for direct access to sensitive personal information. The analysis based on acoustic features reduces privacy concerns and ensures that confidential data remain protected or unidentifiable during the cognitive monitoring process.

Studies examining discourse patterns in participants with ADRD have consistently observed difficulties in word retrieval, less efficient speech, and a notable increase in both the frequency and duration of pauses when their speech is compared to that of healthy adults [35,36]. Notably, in this study, among the features considered crucial for model performance, those related to filled pauses, such as the number of filled pauses and the total time of filled pauses, played a significant role. Filled pauses, such as “um” or “er,” are nonlexical vocalizations. In individuals with dementia, pauses in speech are frequently longer and more frequent, which may indicate challenges with semantic and lexical decision-making, cognitive load, and familiarity with topics [36,37]. This study further highlights that pausing in the speech of individuals with dementia is often considered a dysfluency, serving as a behavioral hallmark that may signify difficulties in social interactions [38]. Our findings are also consistent with previous studies that have examined acoustic-based speech markers in older adults and found good predictive accuracy in identifying those with MCI as compared to being CN [39,40]. Other studies have also found temporal parameters, including prosodic rate and spectrum features, such as Mel-frequency cepstral coefficients, to predict those with MCI or early ADRD [41,42]. These findings offer a research target for further understanding speech issues and mechanisms related to cognitive health. By integrating acoustic analysis into routine clinical assessments, we can potentially enhance current diagnostic tools. This integration provides clinicians with additional quantitative data to support their diagnostic decisions and monitoring of disease progression. Furthermore, the acoustic features identified in this study hold promise for their potential application in large-scale screening programs aimed at identifying individuals at risk of developing MCI. Such screening tools, leveraging these features, could offer a cost-effective and scalable approach, enabling a broader population reach and early intervention strategies. Thus, these findings not only contribute to our scientific understanding but also have practical implications for improving early detection of cognitive impairment.

A unique contribution of our study that has not been well-examined in previous studies is the impact of the speech recording duration on the model performance. Although the full recording yielded the highest AUC (87%), we did not observe substantial differences in model performance based on varying voice recording lengths (eg, 5, 10, 15, and 30 minutes). This finding holds important implications for future studies that involve collecting voice recordings from participants, suggesting that achieving good predictive performance may not require collecting lengthy audio data. It underscores the potential to minimize participant burden and time spent collecting data, while preserving the data's analytical quality. Other strengths of this study include using a community-based sample within a controlled environment for the voice recordings taken during the NP exams. Furthermore, this study uses highly interpretable methods throughout, from feature selection to predictive model construction, achieving good MCI prediction capability. This sets a benchmark for future research attempting more complex analytical approaches. In the future, we can compare complex machine learning methods to fully investigate how to balance the relationship between interpretability and predictive performance.

Important limitations, however, include the inability to account for or investigate the impact of other conditions or risk factors, such as depression [43], that may influence speech patterns within the analysis. Due to the lack of available data on depression at the time of voice recording data collection in FHS, we did not investigate the relationship between depression, cognition, and acoustic features in this study. Future research will be essential to delve into this relationship using more comprehensive cohort data sets. Additionally, our sample consisted mostly of individuals who were White or of European descent, which could potentially limit the generalizability of our findings to other demographic groups. We also recognize that cognition and MCI are not static entities and that individuals with MCI can be considered to be CN at a later point in time [44]. Therefore, it may be possible that some participants were misclassified in terms of their cognitive status in our sample. For example, we acknowledge that the use of the National Institute of Aging–Alzheimer Association (NIA-AA) criteria [45] offers advantages over the National Institute of Neurological and Communicative Disorders and Stroke and the Alzheimer’s Disease and Related Disorders Association and *DSM-IV* criteria, which were used in this study, to ascertain individuals with MCI since it can provide a more comprehensive and inclusive approach, incorporating multiple pathological features. Additionally, the NIA-AA criteria use objective biomarkers and imaging techniques, enhancing diagnostic accuracy and reproducibility. The voice data used in this study were collected in quiet environments, which to some extent limits the widespread applicability of the study results in different environments, such as in-home settings.

To address these limitations, we plan to expand our research in several ways. First, we aim to include more diverse populations in future studies to assess whether the same acoustic features or different ones yield similar results in distinguishing MCI from normal cognition across various demographic groups. Future research should consider using cohorts with biomarker evidence of neurocognitive disorders for further validation of the findings. Additionally, we will explore the inclusion of other medical conditions or factors that may impact model performance, broadening our understanding of how speech patterns can be indicative of cognitive health. Specifically, we recognize that emotions may confound the relationship between speech patterns and cognition. Exploring the detection capability of MCI using voice collected in more real-life environments is another direction for future research. Finally, as we continue to advance in the development of speech-based screening and diagnostic tools, it is crucial to proactively address privacy and data security concerns. While our focus in this paper is primarily on the technical aspects of acoustic feature analysis for cognitive assessment, we recognize the importance of considering the broader societal implications of deploying such technologies in open source or free-market contexts. Safeguards must be implemented to ensure that individuals' privacy rights are respected and that their data are used responsibly and ethically.

### Conclusions

This study demonstrated the potential for accurate identification of MCI using nonsemantic, acoustic speech features. Our research benefits from a well-defined sample and comprehensive speech data collected during NP exams, which have been rigorously analyzed.

## Appendix

Multimedia Appendix 1 Performance of models for MCI prediction using different audio length segments.

## Abbreviations

ADRD: Alzheimer disease and related dementias
AUC: area under the receiver operating characteristic curve
CN: cognitively normal
DSM-IV: Diagnostic and Statistical Manual of Mental Disorders (Fourth Edition)
FHS: Framingham Heart Study
LLD: low-level descriptor
MCI: mild cognitive impairment
NIA-AA: National Institute of Aging–Alzheimer Association
NP: neuropsychological

## Data Availability

The data sets generated during and/or analyzed during this study are available in the Framingham Heart Study [46].
