# Supplementary material for: Detection of Mild Cognitive Impairment From Non-Semantic, Acoustic Voice Features: The Framingham Heart Study
Source: JMIR Aging. 2024 Aug 22;7:e55126. doi: 10.2196/55126 (PMC11377909; doi:10.2196/55126)
Supplement: Multimedia Appendix 1 [file aging_v7i1e55126_app1.docx]

**Table S1.** Performance of models for MCI prediction using different audio length segments.

|  | **Accuracy** | **Sensitivity** | **Specificity** |
| --- | --- | --- | --- |
| 5 mins | 0.74±0.09 | 0.69±0.11 | 0.76±0.09 |
| 10 mins | 0.74±0.11 | 0.69±0.11 | 0.79±0.12 |
| 15 mins | 0.73±0.08 | 0.68±0.10 | 0.77±0.08 |
| 30 mins | 0.78±0.08 | 0.70±0.07 | 0.85±0.09 |
| 1+hour | 0.78±0.13 | 0.72±0.14 | 0.84±0.12 |
